# Response of Onion (*Allium cepa* L.) to nitrogen fertilizer rates and spacing under rain fed condition at Tahtay Koraro, Ethiopia

**DOI:** 10.1038/s41598-018-27762-x

**Published:** 2018-06-22

**Authors:** Kiros Gebretsadik, Nigussie Dechassa

**Affiliations:** 1 Department of Plant Sciences, Aksum University P.o. Box 314, Shire, Ethiopia; 2 Department of Plant Sciences, Haramaya University P.o. Box 138, Dire-Dawa, Ethiopia

## Abstract

Onion is important in the daily Ethiopian diet though the average yield obtained by farmers is very low. This is attributed to a number of constraints among which are poor agronomic practices. Therefore, field experiment was conducted at Tahtay Koraro district to study the effect of nitrogen fertilizer and intra-row spacing on growth and yield of onion. The treatments consisted of a factorial combination of four rates of nitrogen (0, 50, 100 and 150 kg N ha^−1^) and four intra- row spacings (4, 6, 8, and 10 cm). Bombay Red was the variety of onion used in the experiment. The experiment was laid out as RCBD with three replications. The analysis of variance revealed that N and intra-row spacing were significant. Both N and intra-row spacing significantly affected percentage of Bolting plants, leaf length, bulb diameter, and marketable yield. 100 kg N ha^−1^ and a population of 833,300 plants ha^−1^ was found to be the optimum rate to obtain higher marketable bulb yield of 26.72 t ha^−1^ and economically attractive benefits. Therefore, Bombay red variety could be planted at an optimum spacing of 6 cm × 20 cm or 833,300 plant population density ha^−1^ in Tahtay koraro district of northern Ethiopia.

## Introduction

Onion (*Allium cepa* L.) is a vegetable crop grown for its pungent bulbs and flavourful leaves. It belongs to the genus *Allium* of the family *Amaryllidaceae*^1^. Generally, all plant parts of alliums can be consumed by humans except perhaps the seeds^2^. Onions have significant contributions to the nutritional requirements of human beings and have also medicinal values and are primarily consumed for their unique flavour or for their ability to enhance the flavour of other foods^3^.

Over 85.5 million tons of onion was produced worldwide, covering about 4.3 million ha of land, where about 0.57 million ha of the total cultivated land was in Africa^4^. The productivity of onion in Africa is around 14.5 t ha^−1^, which is very low compared to the world average yield of 19.9 t ha^−1^ ^4^. The average bulb yield in the EU is of about 35.3 t ha^−1^ and it is much higher than the world average, indicating the potential yield of onion. Most residents of sub-Saharan Africa including Ethiopia are dependent on rain fed agriculture^5^. Rain fed agriculture in Sub Saharan Africa might remain vital for their food security for some time^6^. About 85% of the population is agrarian and over 90% live on rain fed agriculture^7^.

In Ethiopia about 2.37 million tons of bulbs were produced from 22,035.8 ha of land with an average yield of 10.75 t ha^−1^ ^8^. The Tigray Regional State share was about 7,562 tons from 528.4 ha of land and the average yield was 14.3 t ha^−1^. The potential of Ethiopia for bulb production, for local consumption and export is immense. The yield of onion in Ethiopia is however is low compared to other countries like the Republic of Korea (66.15 t ha^−1^), USA (56.13 t ha^−1^), the Netherlands (51.64 t ha^−1^), Japan (46.64 t ha^−1^) and Egypt (36.16 t ha^−1^)^4^.

Appropriate crop management gives significant contributions to increasing crop yields. Many reports indicate that, in Ethiopia the low productivity of vegetables including onions is attributed to depleted soil fertility and poor agronomic practices such as unbalanced fertilizer application^9,10^. Poor technical knowledge and skill of farmers and development agents in vegetable production are the main problems for the low productivity^11^. The optimum level of agronomic practices like plant population density and fertilizer rate might vary with environment and crop variety^12^. Thus, it is very difficult to give general recommendations applicable to the different agro ecological zones. But to optimize onion productivity a full package of information is required for specific growing system^13,9^. Optimum plant population density has a dual advantage. It avoids strong competition between plants for growth factors such as water, nutrients, and light while enabling efficient use of available of land resources^14^.

Higher yield and better control of over or under-sized bulbs could be obtained if plants are grown at optimum density. The control of plant spacing is therefore a valuable way of controlling bulb size, shape, and yield^15^. There is however lack of improved techniques for onion production in northern Ethiopia and the issue was serious particularly in the study area. This research, has therefore, the objective to determine the impact of nitrogen fertilizer and spacing on growth and yield of onion.

## Methodology

### Description of the study area

**Figure 1:**
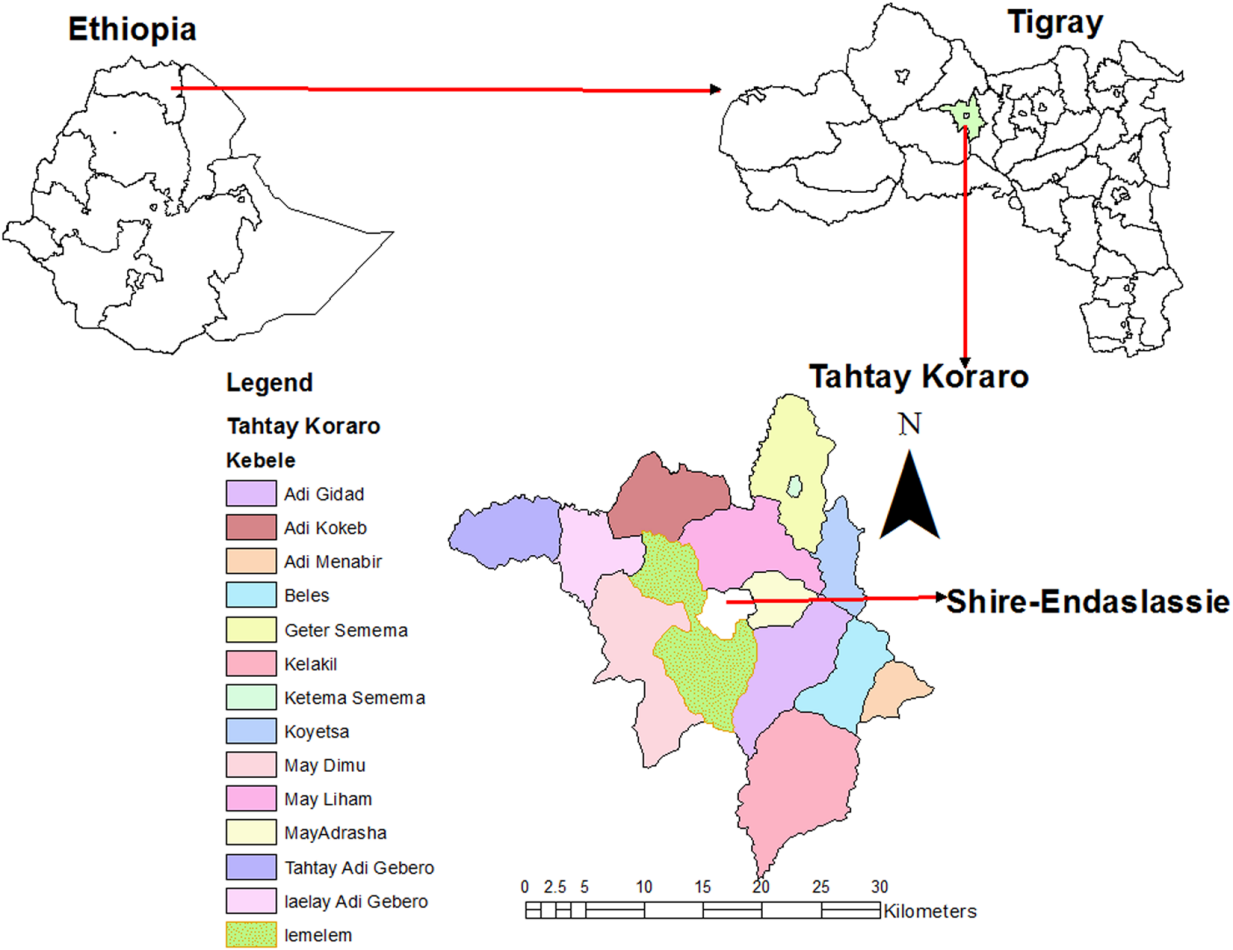
Geographical location of the study area; Tahtay Koraro district, in Tigray Regional State, Northern Ethiopia.

The study was conducted at Lemlem peasants’ association at the Well Foundation demonstration farm near Shire-Endaslassie town, which is part of Tahtay Koraro district in Tigray Regional State of Ethiopia. It is located at an altitude of 1900 m above sea level (Fig. 1). The site is situated at latitude of 14°6′N and longitude of 38°17′E. The mean annual rainfall is 990 mm and average annual minimum and maximum temperatures are 12.4° C and 28.5° C, respectively. The rainy season extends from May to September with maximum rain received in the months of June to August.

There is little information about soil type and its corresponding biological and physico-chemical properties of the study area. Hence, the pre-planting soil samples collected were analyzed in Mekele Regional Soil laboratory results for some selected physical and chemical properties of the study area. Analysis revealed that the field was sandy clay loam soil with pH of 6.57, organic carbon 1.29%, total nitrogen 0.08%, available phosphorus 43.62 ppm, exchangeable potassium of 1.55 cmol (+) kg^−1^ and CEC of 14.93 cmol kg^−1^ soil (Table 1). The rural area around the study site is known for the mixed crop-livestock farming system^16^.

**Table 1:** Soil Physico-chemical properties of the experimental site before planting.

Soil property	Result	Type (Quality)
Soil particle size (%)		
Sand	67.20%	
silt and	11.30%	
clay	21.50%	
Textural class	Sandy clay loam	
pH	6.57	Neutral in reaction
Total N (%)	0.08	Very low or deficient
Organic Carbon (%)	1.29	Moderate
Organic matter %	2.23	Moderate
Exchangeable K (cmol (+)) kg^−1^	1.55	High
Available P (ppm)	43.62	Very high
EC (dSm^−1^)	0.225	Non-saline
CEC (cmol kg^−1^ soil)	14.93	Moderate

Source: Mekelle Soil laboratory of Tigray Regional Soil Laboratory.

### Description of experimental materials

The onion (*Allium cepa* L.) variety Bombay Red was used for the experiment. The seeds were obtained from Melkassa Agricultural Research Centre. The variety was released by the research centre in 1980. The cultivar is adapted to altitudes ranging from 700 to 2000 m above sea level. It has flat globe-shaped medium bulbs^17^. Bombay Red is an early maturing Variety for dry and warmer conditions producing small to medium-sized bulbs, which are globe-shaped, purplish red and pungent. They are valued for their earliness in East Africa^2^. The source of the nitrogen fertilizer was urea (46% N).

### Treatments and experimental design

The treatments consisted of four intra-row plant spacing (4, 6, 8, 10 cm, and 20 cm spacing between rows), which form 1250000, 833300, 625000 and 500000 plant population densities per hectare, respectively (Table 2) and four levels of nitrogen levels (0, 50, 100 and 150 kg N ha^−1^). The experiment was laid out as a randomized complete block design (RCBD) in a 4 × 4 factorial arrangement and replicated three times.

**Table 2:** Treatment combinations of fertilizer rates, plant spacing and plant populations.

Nitrogen Rate (kg ha^−1^)	Intra row Spacing (m)	Plant Spacing (m)	Equivalent area (m^2^)	Plants per m^2^	Plants ha^−1^
0	0.10	0.10 × 0.20	0.020	50.0	500,000
	0.08	0.08 × 0.20	0.016	62.5	625,000
	0.06	0.06 × 0.20	0.012	83.3	833,300
	0.04	0.04 × 0.20	0.008	125.0	1,250,000
50	0.10	0.10 × 0.20	0.020	50.0	500,000
	0.08	0.08 × 0.20	0.016	62.5	625,000
	0.06	0.06 × 0.20	0.012	83.3	833,300
	0.04	0.04 × 0.20	0.008	125.0	1,250,000
100	0.10	0.10 × 0.20	0.020	50.0	500,000
	0.08	0.08 × 0.20	0.016	62.5	625,000
	0.06	0.06 × 0.20	0.012	83.3	833,300
	0.04	0.04 × 0.20	0.008	125.0	1,250,000
150	0.10	0.10 × 0.20	0.020	50.0	500,000
	0.08	0.08 × 0.20	0.016	62.5	625,000
	0.06	0.06 × 0.20	0.012	83.3	833,300
	0.04	0.04 × 0.20	0.008	125.0	1,250,000

0.1 m, 0.08 m, 0.06 m and 0.04 m are the intra row spacing when multiplied each by 0.2 m common inter row spacing provides 500000, 625000, 833300 and 1250000 plant population density ha^−1^.

Treatments were assigned to each plot within each block randomly. The total numbers of plots were 48. The distance between adjacent plots and blocks were 0.75 m and 1 m, respectively. Each plot included 11 rows. All other cultural practices such as weeding, supplementary irrigation control of pests and diseases were performed in compliance with the regional recommendations. Data were collected from plants in the central rows, leaving aside plants in the outer most rows as well as those at the end of each row, to avoid edge effects. At physiological maturity when 70% of their leaves senesced, plants were harvested and used for determining yield: bolting, leaf length, number of leaves per plant, stand count, neck diameter and marketable yield.

### Statistical analysis

Data were subjected to analysis of variance using SAS Statistical Software package version 9.1^18^. Means that differed significantly were separated using the Least Significant Difference (LSD) test procedure at 5% level of significance. Pearson Correlation coefficients were determined for parameters using the same software.

### Economic analysis

The economic analysis was computed using the procedure described by CIMMYT^18^ to identify economically attractive combination of nitrogen fertilizer and intra-row spacing. From the final experimental data, the average marketable yield of 16 treatments was obtained that was adjusted down wards by 10% as farmers using the same technologies would obtain yields 10% lower than the yields obtained by researchers^19^. This was due to the fact that under experimental condition there was better crop management and small plot size. To obtain the gross field benefits it is essential to know the market price which is the value of one kg of onion bulb at harvest time. Adjusted yield multiplied by field price gives gross field benefit.

The cost and benefits were calculated for each treatment. Purchasing cost for Urea is taken as 11 Birr kg^−1^ and cost of daily labour is taken as 50 Birr day^−1^. The selling price of onion at the local market was 9 Birr. The variable cost of fertilizer and labour cost for application of fertilizer and transplanting seedlings for each treatment was reduced from gross benefit. Other costs that do not vary among all treatments (like ploughing, weeding, harvesting, etc.) were not included as variable costs.

$$\begin{array}{rcl}{\rm{Total}}\,{\rm{variable}}\,{\rm{cost}}\,({\rm{Birr}}) & = & {\rm{cost}}\,{\rm{of}}\,{\rm{fertilizer}}\,{\rm{and}}\,{\rm{labour}}\,{\rm{cost}}\,\\  &  & {\rm{for}}\,{\rm{application}}\,{\rm{of}}\,{\rm{fertilizer}}\,{\rm{and}}\\  &  & {\rm{transplanting}}\,{\rm{seedlings}}\,{\rm{for}}\,{\rm{each}}\,{\rm{treatment}}\\ {\rm{Gross}}\,{\rm{field}}\,{\rm{benefit}} & = & {\rm{Adjusted}}\,{\rm{yield}}\times {\rm{unit}}\,{\rm{price}}\,{\rm{of}}\,{\rm{onion}}\\ {\rm{Net}}\,{\rm{benefit}} & = & {\rm{Gross}}\,{\rm{benefit}}-{\rm{total}}\,{\rm{variable}}\,{\rm{cost}}\end{array}$$

MRR analysis was carried out on undominated treatments in a stepwise manner and minimum marginal rate of return was take as 100%, as recommended especially for poor farmers in developing countries or for technologies requiring substantial change to a farming system^20^ and^21^.

## Result and Discussion

### Bolting Percentage

The analysis of variance showed that bolting percentage was highly significantly (P < 0.01) influenced by the main effect of nitrogen, but it was not affected by the main effect of intra-row spacing (Table 3).

**Table 3:** The effects of nitrogen and intra row spacing on plant height, bolting percentage, leaf length, number of leaves per plant, field stand count, neck diameter and marketable yield.

Nitrogen (kg ha^−1^)	Plant height after 30 days (cm)	Bolting percentage (%)	Leaf length (cm)	Number of leaves/plant	Field stand count (%)	Neck diameter (cm)	Marketable yield (t ha^−1^)
0	33.917	5.408	44.142	7.833	94.089	0.937	16.588
50	34.917	3.503	44.692	6.667	94.360	0.988	24.114
100	34.883	2.290	46.467	8.667	94.301	1.046	31.455
150	34.458	2.065	52.141	8.333	94.479	1.210	29.669
F-test	Ns	*	*	**	Ns	*	**
LSD(0.05)		0.639	5.362	0.783		0.115	2.097
Intra-row Spacing (cm)							
4	34.783	3.305	46.373	6.583	91.286	1.080	24.23
6	34.380	3.293	47.360	8.167	94.169	1.055	26.72
8	34.442	3.340	46.892	8.167	94.361	0.998	25.43
10	34.575	3.328	46.817	8.583	97.413	1.049	25.45
F-test	Ns	Ns	Ns	**	*	Ns	*
LSD(0.05)				0.783	2.71		2.097
CV (%)	3.771	23.115	13.724	11.925	3.446	13.204	9.882

Means in columns sharing a common letter are not significantly different at 5% level of significance Ns = non-significant. *^,^**, significant at 5% and 1% respectively.

Bolting percentage decreased with the increase in the rate of nitrogen application. In response to increasing the rate of nitrogen from 0 to 50 kg N ha^−1^ bolting percentage was decreased by 35%. When the rate of nitrogen increased further from 50 to 100 kg N ha^−1^, bolting percentage again dropped by 35%. However, increasing the rate of nitrogen from 100 to 150 kg N ha^−1^ did not change bolting percentage (Table 3). In general, increasing nitrogen from the 0 to 150 kg N ha^−1^ decreased bolting percentage by 62%. This result is in line with a field research reported that, nitrogen fertilization significantly reduced bolting in onion where the proportion of bolters per plot decreased by about 11 and 22% in response to application of nitrogen at 69 and 92 kg N ha^−1^, respectively over the control treatment^22^. This could be associated with the effect of N in extending the vegetative growth period of plants while delaying flowering. This can duet to the fact that low nitrogen promotes bolting in onion^23^.

The C/N ratio determines whether the onion plant remains vegetative or processes a flower stalk^24^. It was found that bulb N content increased with N fertilization and bolting decreased seedily with increasing bulb and shoot N contents^25^. This indicates that increasing N fertilization rates possibly decreased onion plant’s C/N ratio which could partly explain results in this study.

Although this study did not show significant difference in bolting percentage due to intra row spacing, some research results revealed significant differences among plants grown at different intra-row spacing in bolting percentage. Closer spacing of 5 × 20 cm increased percentage of bolters by 14.5%, while nitrogen application decreased the incidence of bolting^26^. This may be due to the fact that, competition among plants in densely populations to result in smaller bulbs that are less susceptible to bolting.

### Leaf length

The analysis of variance showed that the main effect of nitrogen fertilization significantly increased leaf length of onion plants. However, leaf length was not significantly influenced by intra-row spacing (Table 3). Increasing the rate of nitrogen from 0 to 100 kg N ha^−1^ did not change the leaf length of onion. However, in response to increasing nitrogen from 0 to 150 kg N ha^−1^, leaf length increased significantly by 18% or 8 cm. The result is consistent with previous research findings reporting that the higher nitrogen rates resulted in longer leaves of onion^15^. The increase in leaf length of onion in response to the increased rate of nitrogen application may be attributed to the positive effect of nitrogen on vegetative growth and leaf expansion^27^. Consistent with this research result^28^, found that nitrogen fertilization not only promotes leaf growth but also enhances efficient uptake and utilization of other nutrients especially phosphorus and potassium.

The analysis of variance of plant height 30 days after transplanting shows that the application of different rates of nitrogen and intra-row spacing had no any significant (P < 0.05) effect on plant height. This may be due to the fact that the competition for resources at the early stage of young seedlings may be minimum.

### Number of leaves per plant

Increasing the rate of nitrogen from 0 to 50 kg N ha^−1^ significantly decreased leaf number (Table 3). However, increasing the rate further from 50 to 100 kg N ha^−1^ increased leaf number significantly by about 29%. Increasing the rate of nitrogen beyond this level did not however affect the number of leaves produced by the onion plant. The decrease in the number of leaves with the increase of nitrogen rate from 0 to 50 kg N ha^−1^ is difficult to explain, but could be attributed to other factors. However, increase in the number of leaves with further increase in the rate of nitrogen could be attributed to enhanced photo-assimilate production and cell division, and vegetative growth in response to the enhanced sappy of the nutrient^27^. This shows that, nitrogen played an important role in leaf production and vigorous vegetative growth. Similar results were previously reported^29^.

The analysis further revealed that widening the intra-row spacing from 4 to 6 cm significantly increased the leaf number of onions. However, widening the space beyond 6 cm did not lead to significant differences in leaf number produced per plant (Table 3). The leaf number obtained in response to spacing the plants 6 cm apart exceeded the leaf number of plants spaced 4 cm apart by 24%. This increase in the number of leaves with widening of plant spacing may be attributed to ease of competition among plants for growth resources. Corroborating the result of this study, research result for onion indicated that the greatest number of leaves per plant was found in the widest row spacing^30^. This may be due to the fact that, wider spacing has low competition for resources to increase leaf number production and vigorous vegetative growth. This result is, however, unlike with the findings of a researcher who reported on onion crop that more leaves were produced at lower planting density and lower leaf number at the higher planting density^31^. This may due to the differences in environmental factors such as the soil nutrient content, temperature, rainfall and also management practices between the experimental sites.

### Field Stand Count

The analysis of variance showed that neither the main effect of nitrogen nor the interaction effect of nitrogen and intra-row spacing affected field stand count of the onion plants. However, the analysis showed that field stand count was affected significantly by the main effect of intra-row spacing (Table 3).

The plants grown in the wider spaced plots (10 cm × 20 cm) performed well, 97.4% of them being able to survive whereas plants grown in the narrowly spaced plots (4 cm × 20 cm) had almost 9% total plant population loss (Table 3). There is no significant difference between 6 cm and 8 cm intra-row spacing on field stand count of onion plants. As the intra raw-spacing increased from 6 cm or/and 8 cm to 10 cm, plant loss decreased by about 3%. This shows that narrowly spaced plants face severe competition compared with the wider spaced plants as competition for resources such as nutrients, moisture, and sun light etc causes plant losses. This may be due to inter-plant competition become too severe as intra-row spacing decreases and plant losses also increase during sever competition.

### Neck diameter

The ANOVA showed that neck diameter was significantly influenced by the main effect of nitrogen, but not by the main effect of intra-row spacing or by the interaction effect of nitrogen and intra-row spacing (Table 3). Increasing the rate of nitrogen from 0 to 50 and 50 to 100 kg N ha^−1^ did not affect neck diameter. However, increasing the rate of nitrogen from nil to 150 kg N ha^−1^ increased neck diameter by 28%. This may be due to direct effect of nitrogen on neck diameter thickening and could imply that higher doses of nitrogen reduce shelf life of onion bulb. Neck diameter (thickness) in onion is an important character, because it indicates bulb storage ability. The onion with thin neck diameter, store better than thick diameter^32^. The increase in neck diameter might show its involvement in the synthesis of amino acids, as they link together and form proteins and make up metabolic processes required for plant growth including neck thickening. This result is in agreement with that of Jilani who reported that application of N at the rate of 200 kg ha^−1^ increased the number of thick-necked bulbs^15^.

Although this study did not show any significant difference using intra-row spacing in this parameter, some research in Haryana, India found that the highest density (10 × 5 cm) had the thinnest necks compared with the medium (10 × 7.5 cm) and low (10 × 10 cm) densities^33^.

### Marketable yield

The main effect of nitrogen significantly (P < 0.01) influenced marketable bulb yield (Table 3). However, the main effect of intra-row spacing did not influence this parameter (Table 3). Marketable yield varied from 16.588 to 31.455 t ha^−1^, the latter being recorded at 100 kg N ha^−1^. Increasing the rate of nitrogen from 0 to 50 kg N ha^−1^ markedly increased marketable fresh bulb yield by 45%. Increasing the rate of nitrogen from 50 to 100 kg N ha^−1^ increased the marketable fresh bulb yield by a further percentage of 30%. However, increasing the rate of nitrogen from 100 to 150 kg N ha^−1^ decreased marketable fresh bulb yield by 5% (Table 2). Therefore, increasing the rate of nitrogen from 0 to 100 kg N ha^−1^ increased fresh bulb yield by 85%. Thus, 100 kg N ha^−1^ gave the optimum fresh marketable bulb yield, and there is no need to increase the rate of the nitrogen above this rate to produce the crop. The increment in marketable fresh bulb yield due to the application of nitrogen may be attributed to an increase in leaf area index, bulb diameter, and average bulb weight^29^.

Different researchers reported bulb yield improvement in response to N fertilization. There are reports indicating that different plant growth characters (plant height and bulb diameter) are known to increase the yield of onion^29^. Consistent with the results of this study, an experiment carried out on onions (*Allium cepa*) using nitrogen rates ranging from 0 to 180 kg ha^−1^ revealed that the highest yield and percentage of marketable bulb yield was found at 60 kg N ha^−1^ with the 180 kg N ha^−1^ resulting in yield that was even lower than that of the treatment without nitrogen^34^.

Widening the intra-row spacing from 4 to 6 cm significantly increased the fresh marketable bulb yield by 10%. However, widening the intra-row spacing to the highest levels did not change marketable fresh bulb yield (Table 3). This result shows that plants grown at the wider spacing of 6, 8, and 10 cm produced the highest fresh marketable bulb yields due to lesser competition among them than plants grown at the narrowest intra-spacing of 4 cm. In contrast to this study, onion Bombay Red variety produced in the East African great valley gave the highest marketable bulb yield at the narrower spacing of 4 cm compared to the relatively wider spacings of 8 cm and 10 cm^35^. Moreover, other researchers^36^ reported the interaction between intra-row spacing and planting time on onion yield. Since there is scarcity of resources such as land, fertilizers, irrigation water, using wider spacing between onion plants may not give optimum yields and economic benefits.

### Cost benefit analysis

The cost benefit analysis revealed that, the highest net benefit of 266028 Birr (Ethiopian Currency) was obtained by application of 100 Nkg ha^−1^ at intra row spacing of 6 cm followed by the net benefit of Birr 256206, which was obtained using 100 N kg ha^−1^ at intra row spacing of 10 cm (Table 4). The lowest net benefit, Birr 128394, was obtained from the no N control with 10 cm intra row spacing. Moreover, the dominance analysis in Table 3 showed that 9 of the 16 treatments were dominated. Dominated treatments were off the comparison for marginal analysis. Calculation of net benefit accounts for costs that vary but also it is important to compare the extra or marginal costs with the extra or marginal net benefits. The procedure of calculating the marginal rates return (MRR) of alternative treatments, proceeding in step wise from the least costly treatment to the next costliest treatment and deciding if they are acceptable to farmers is known as marginal analysis^19^.

**Table 4:** Partial budget analysis.

Treatments		Marketable yield (t ha^−1^)	Adjusted yield (t ha^−1^)	Gross field benefit (Birr ha^−1^)	Total VC (Birr)	Net Benefit (Birr)	Dominance*
N (kg ha^−1^)	Spacing(cm)						
0	10	15.88	14.292	128628	234	128394	Undominated
	8	15.98	14.382	129438	305	129133	Undominated
	6	15.97	14.373	129357	375	128982	Dominated
	4	18.52	16.668	150012	429	149583	Undominated
50	10	24.42	21.978	197802	1573	196229	Undominated
	8	24.16	21.744	195696	1644	194052	Dominated
	6	26.50	23.85	214650	1714	212936	Undominated
	4	21.38	19.242	173178	1768	171410	Dominated
100	10	31.99	28.791	259119	2913	256206	Undominated
	8	31.86	28.674	258066	2984	255082	Dominated
	6	33.22	29.898	269082	3054	266028	Undominated
	4	28.75	25.875	232875	3108	229767	Dominated
150	10	29.53	26.577	239193	4252	234941	Dominated
	8	29.71	26.739	240651	4323	236328	Dominated
	6	31.18	28.062	252558	4393	248165	Dominated
	4	28.26	25.434	228906	4447	224459	Dominated

^*^When the new technology surpassed the conventional practice, it is said to be Undominated. When the new technology yields lower benefit then the technology is indicated as dominated.

The result of the marginal analysis is presented in Table 5. Based on the analysis, all of the results showed more than the minimum rate of return (100%). Further comparison was made depending on the MMR% and net benefit between the treatments. Recommendations may not necessarily be based on the highest marginal rate of return though. Because farmers who use onion using intra-row spacing of 8 cm and no nitrogen highest rate of return (16491.9%) was obtained, but if farmers stopped there, they would miss the opportunity of further earnings, an attractive rate of returns by investing 100 kg of nitrogen fertilizer and reducing intra row spacing from 8 cm to 6 cm. Farmers should continue investing as long as the returns to each extra unit invested (measured by the marginal rate of return) are higher than the cost of extra unit invested (measured by the minimum acceptable rate of return)^19^.

**Table 5:** Analysis of marginal rate of return.

Treatments		Adjusted Marketable yield (tha^−1^)	Net Benefit (Birr)	Total variable Cost (Birr)	Marginal increase net benefit (Birr)	Marginal increase variable Cost (Birr)	MRR %
N (kg ha^−1^)	Spacing (cm)						
0	10	14.292	128394	234	739	71	1040.9
0	8	14.382	129133	305	20450	124	16491.9
0	4	18.52	149583	429	46646	1144	4077.5
50	10	24.42	196229	1573	16707	141	11848.9
50	6	26.50	212936	1714	43270	1199	3608.9
100	10	31.99	256206	2913	9822	141	6966.0
100	6	33.22	266028	3054			

MRR is calculated by dividing the marginal increase in net benefit with the marginal increase in variable cost and multiplied by 100.

MRR implies what a producer can get to receive by switching technologies from the farmers practice to the improved new one, hence, 6966% MRR indicates that by investing 1 Birr a farmer can get 69.66 Birr by using combination of 100 N kgha^−1^ and 6 cm intra row spacing which is above the minimum rate of return and produced the highest net benefit. High bulb yield (33.22 t ha^−1^) was obtained at this treatment. High net benefit from the foregoing treatments could be attributed to high yield and the low net benefit was attributed to low yield.

Thus, 100 N ha^−1^ combined with 6 cm intra-row spacing could be recommended and 10 cm combined with 100 kg N ha^−1^ and 100 kg N ha^−1^ combined with 10 cm as second and third alternatives. High bulb yield and low cost evidently leads to maximum income. Therefore, it is advisable to apply 100 kg nitrogen with intra-row spacing of 6 cm to get high profit in onion production under rain-fed condition in the study area.

## Conclusion

Onion is commonly produced using irrigation in Ethiopia but it is also possible to produce under rain fed condition to maintain higher yield as far as the soil nutrient and spacing is maintained. Combining optimum nitrogen fertilizer rate and intra row spacing helps to get higher yield and higher economic benefit. From the results of this investigation, it can be concluded that, nitrogen application at the rate of 100 kg N ha^−1^ combined with intra row spacing of 6 cm and inter-row spacing of 20 cm which helps to produce about 833,300 plants ha^−1^ is optimum rate to earn maximum marketable yield and higher economic profit from Bombay Red cultivar in rain fed areas like Tahtay Koraro district or Shire area, northern Ethiopia.
